# Impact of the MLC leaf‐tip model in a commercial TPS: Dose calculation limitations and IROC‐H phantom failures

**DOI:** 10.1002/acm2.12819

**Published:** 2020-01-21

**Authors:** Brandon Koger, Ryan Price, Da Wang, Dolla Toomeh, Sarah Geneser, Eric Ford

**Affiliations:** ^1^ Department of Radiation Oncology University of Washington School of Medicine Seattle WA USA

**Keywords:** MLC leaf tip, multi‐leaf collimator, treatment planning, treatment planning system validation

## Abstract

**Purpose:**

Treatment planning system (TPS) dose calculation is sensitive to multileaf collimator (MLC) modeling, especially when treating with intensity‐modulated radiation therapy (IMRT) or VMAT. This study investigates the dosimetric impact of the MLC leaf‐tip model in a commercial TPS (RayStation v.6.1). The detectability of modeling errors was assessed through both measurements with an anthropomorphic head‐and‐neck phantom and patient‐specific IMRT QA using a 3D diode array.

**Methods and Materials:**

An Agility MLC (Elekta Inc.) was commissioned in RayStation. Nine IMRT and VMAT plans were optimized to treat the head‐and‐neck phantom from the Imaging and Radiation Oncology Core Houston branch (IROC‐H). Dose distributions for each plan were re‐calculated on 27 beam models, varying leaf‐tip width (2.0, 4.5, and 6.5 mm) and leaf‐tip offset (−2.0 to +2.0 mm) values. Doses were compared to phantom TLD measurements. Patient‐specific IMRT QA was performed, and receiver‐operating characteristic (ROC) analysis was performed to determine the detectability of modeling errors.

**Results:**

Dose calculations were very sensitive to leaf‐tip offset values. Offsets of ±1.0 mm resulted in dose differences up to 10% and 15% in the PTV and spinal cord TLDs respectively. Offsets of ±2.0 mm caused dose deviations up to 50% in the spinal cord TLD. Patient‐specific IMRT QA could not reliably detect these deviations, with an ROC area under the curve (AUC) value of 0.537 for a ±1.0 mm change in leaf‐tip offset, corresponding to >7% dose deviation. Leaf‐tip width had a modest dosimetric impact with <2% and 5.6% differences in the PTV and spinal cord TLDs respectively.

**Conclusions:**

Small changes in the MLC leaf‐tip offset in this TPS model can cause large changes in the calculated dose for IMRT and VMAT plans that are difficult to identify through either dose curves or standard patient‐specific IMRT QA. These results may, in part, explain the reported high failure rate of IROC‐H phantom tests.

## 1 INTRODUCTION

Installation of a new treatment planning system (TPS) requires rigorous commissioning and validation testing to ensure dose calculation accuracy.^1^ This includes external validation of the dosimetry as recommended by various national and international groups^1^, ^2^, ^3^, ^4^ and as required for clinical trials.^5^ Intensity‐modulated radiation therapy (IMRT) credentialing is offered by the Imaging and Radiation Oncology Core Houston branch (IROC‐H) in the form of anthropomorphic phantoms for several anatomical sites.^6^ Recent results from IROC‐H show that 17% of institutions using the service had considerable dose calculation errors in their TPS^7^ and that patient‐specific IMRT quality assurance (QA) at many of these institutions failed to predict these errors.^8^ Despite significant evidence that patient‐specific IMRT QA fails to detect TPS errors,^9^, ^10^, ^11^, ^12^ many institutions continue to rely on it for TPS commissioning and validation.

Although these failure patterns are established and appreciated in the community, it is not well‐known which specific factors within the TPS may cause these failures, especially for newer planning systems. Small changes in the multileaf collimator (MLC) position are known to translate into large dose deviations in IMRT plans.^13^, ^14^, ^15^, ^16^ Previous studies have examined the potential impact of MLC miscalibration both in delivery^17^ and as seen in log files.^18^ Studies have also examined the dosimetric leaf gap (DLG) parameter used in the Eclipse TPS (Varian Medical Systems, Palo Alto, CA) to describe leaf offset positions, showing potentially large dosimetric impacts.^19^, ^20^, ^21^ However, these studies do not explore whether these results translate across planning systems, nor do they assess the detectability of these deviations with QA devices or whether anthropomorphic phantom tests may aid in the detection of inaccurate MLC modeling. Furthermore, the RayStation TPS (RaySearch Laboratories, Stockholm, Sweden) MLC model includes several unique parameters that have not been extensively investigated.

The purpose of this study was to investigate the dosimetric impact of parameters in the MLC model in a commercial TPS. Additionally, the detectability of suboptimal MLC modeling was assessed through two common tests: measurements with a patient‐specific IMRT QA device and validation with a third‐party anthropomorphic phantom.

## 2 METHODS AND MATERIALS

### 2.1 IROC‐H phantom irradiation and TPS beam model

Photon planning in RayStation v.6.1 was commissioned and validated following the most recent recommendations of AAPM Medical Physics Practice Guideline 5.a.^1^ All recommended validation tests from AAPM's MPPG 5.a^1^ and TG‐119^22^ were performed and were found to agree to within the recommended tolerances.

**Figure 1:**
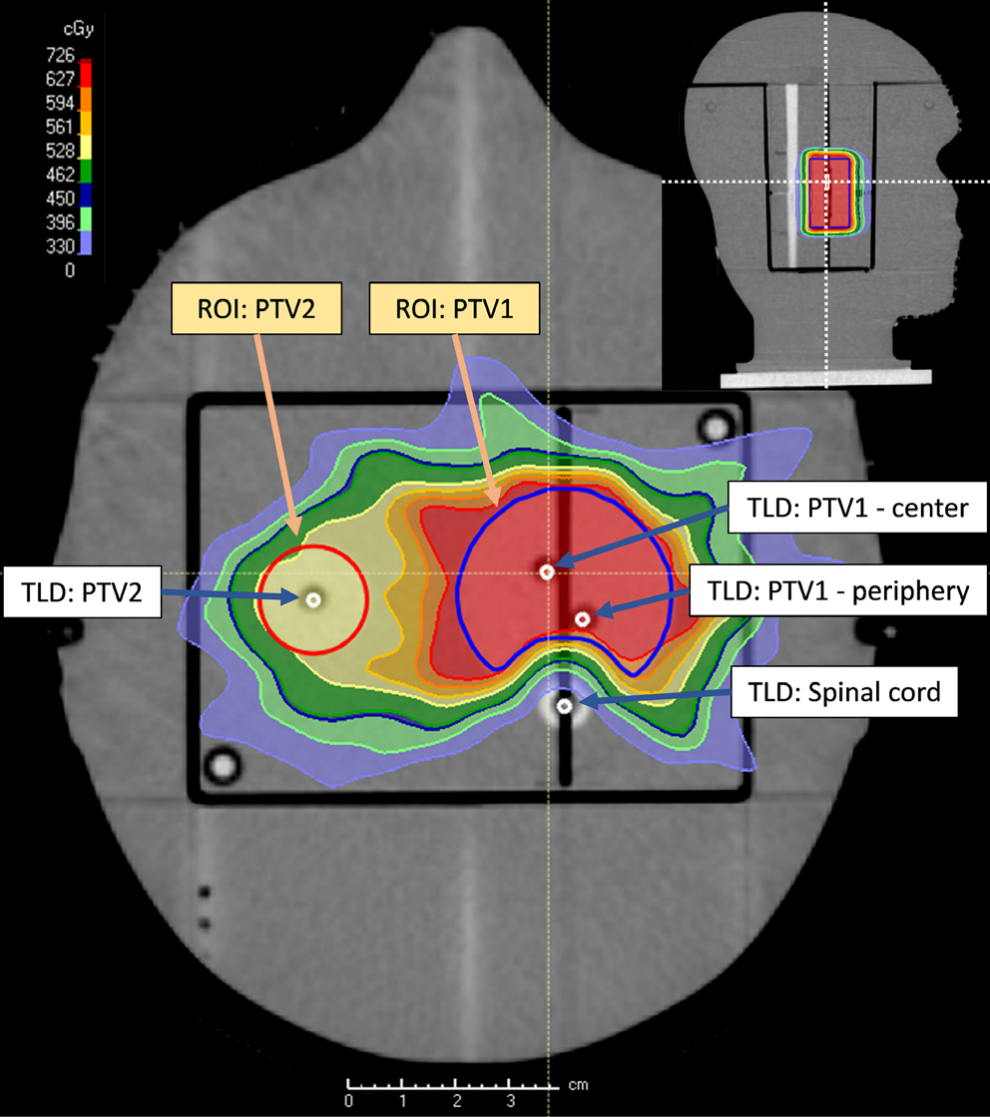
Schematic of Imaging and Radiation Oncology Core Houston (IROC‐H) head‐and‐neck phantom. Superior thermoluminesent dosimeters (TLDs) are shown and labeled.

External validation was carried out through irradiation of the IROC‐H head‐and‐neck phantom.^23^ Such tests are recommended by MPPG 5.a^1^ and other reports.^2^, ^3^, ^4^ This anthropomorphic head‐and‐neck phantom (Figure 1) contains planning structures and dosimeters (thermoluminesent dosimeters [TLDs] and film) and provides rigorous testing of the accuracy of the TPS model. The phantom contains eight total TLDs, labeled here as PTV1 center, PTV1 periphery, PTV2, and Spinal cord, each with both superior and inferior TLDs. For simplicity and clarity, this study only includes the superior TLDs, as doses from the superior and inferior TLDs were found to agree within 0.3% in the PTVs and 1.5% in the spinal cord.

The phantom was scanned on a CT simulator following standard clinical workflow, and nine uniquely optimized treatment plans were created in the TPS following the planning guidelines given by IROC‐H. Of these plans, five plans were step‐and‐shoot IMRT plans containing either seven or nine beams evenly distributed around the patient. Note that Elekta linear accelerators do not allow dynamic IMRT beam delivery except for VMAT plans. The four remaining plans were delivered using VMAT containing either two or three full arcs with variable collimator angles, dose rate, and gantry speed. The total monitor units (MU) for all plans ranged between 1469 and 2114 MU. Table 1 summarizes beam arrangement and MU information for each plan. All plans were optimized by prioritizing different optimization objectives, while still remaining within the IROC‐H planning guidelines, in order to obtain unique optimization solutions. All plans were typical of how our institution would treat a head‐and‐neck patient, both in terms of complexity and beam arrangements.

**Table 1:** Description of the treatment plans used in this study.

Plan name	Number of beams	Total MU	Comments	
IMRT0	7	1542	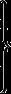	Step‐and shoot delivery Equally spaced beams
IMRT1	7	1772		
IMRT2	7	1733		
IMRT3	9	1747		
IMRT4	9	1818		
VMAT1	2	1711	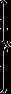	Full arcs Variable dose rate
VMAT2	2	1871		
VMAT3	2	2114		
VMAT4	3	1469		

Five step‐and‐shoot IMRT plans and four full arc VMAT plans were individually optimized to ensure a variety of plans with unique solutions were investigated.

One of these plans, a 7‐field step‐and‐shoot IMRT plan (Plan IMRT0 in Table 1), was delivered to the IROC‐H phantom after standard patient‐specific QA and physics checks were performed. Despite extensive in‐house validation following vendor recommendations and AAPM standards, the results failed the ±7% criteria set by IROC‐H. Specifically, the dose deviation for each TLD (IROC‐H measured vs. TPS dose) was: PTV1 center: −7%, PTV1 periphery: −9%, PTV2: −4%, Spinal cord: −4%. Partially based on these findings, it was found that small adjustments in two parameters — the MLC leaf‐tip width and leaf‐tip offset — caused large variations in TPS‐calculated dose for IMRT and VMAT plans with minimal apparent effect on open fields. This effect was particularly prominent for the leaf‐tip offset.

**Figure 2:**
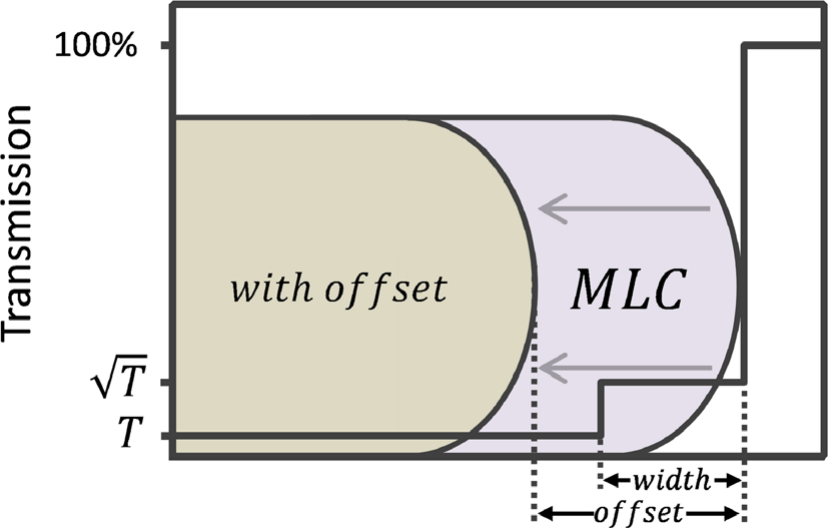
Illustration of the multileaf collimator (MLC) model in the treatment planning system (TPS). Two parameters are used: (1) leaf‐tip width, which defines a region of partial transmission, and (2) leaf‐tip offset, which defines a shift in the leaf from its nominal position and is represented by the leaf shown in brown. The solid black line represents the percentage transmission through the leaf.

In the RayStation TPS, the leaf is modeled as a double step function (Figure 2). The leaf‐tip width is defined as the width of the region of the MLC that has partial transmission, modeled with a transmission of $\sqrt{T}$, where T is the intraleaf leakage. The leaf‐tip offset is defined as the offset of the MLC from the nominal position. A positive leaf‐tip offset models an effectively larger field size, while a negative leaf‐tip offset models a smaller field size. The leaf‐tip offset is somewhat similar to a parameter used in the Eclipse TPS, the dosimetric leaf‐gap (DLG),^20^ which is defined as the difference between the 50% field width and the nominal radiological field size.

### 2.2 Simulated beam model deviations and detectability with QA

Twenty‐seven inaccurate beam models were created using combinations of leaf‐tip offset (ranging from −2.0 to +2.0 mm at 0.5 mm intervals) and leaf‐tip width (2.5, 4.5, and 6.0 mm). Leaf‐tip values of approximately −1 to +1 mm led to beam models that were very similar when profile and PDD data and IMRT QA were compared but could be distinguished based on IROC‐H TLD measurements. The range of leaf‐tip offset values was chosen to encompass these values with some margin on either end. Each of the nine plans previously mentioned were recalculated, but not re‐optimized, on each beam model. Mean doses to each of the TLDs within the phantom were extracted from the TPS and compared against both the IROC‐H‐measured doses and the TLD dose as calculated by the TPS using the clinical model.

To determine whether the failures in the IROC‐H phantom delivery would be detectable through patient‐specific IMRT QA, each plan was delivered to a 3D diode array (ArcCheck, Sun Nuclear Inc, Melbourne, FL). The delivered plan was compared to the plans calculated with models using different leaf‐tip offset values. Gamma analysis^24^ was performed using a low‐dose threshold of 10% and criteria of both 2% global/2 mm and 3% global/3 mm.

Receiver‐operating characteristic (ROC) curves, often used in medicine to assess the detectability of a given end point,^9^, ^25^ were generated by varying the gamma pass rate. They plot the true positive rate (sensitivity) versus the false positive rate (1‐specificity) of a given test. Here, plans calculated on the clinical model were considered passing, while plans calculated on other beam models were considered failing. Test quality can be quantified as the area under the curve (AUC), ranging from 0.5 (poor) to 1.0 (excellent).

## 3 RESULTS

### 3.1 TPS model and agreement with IROC‐H

**Figure 3:**
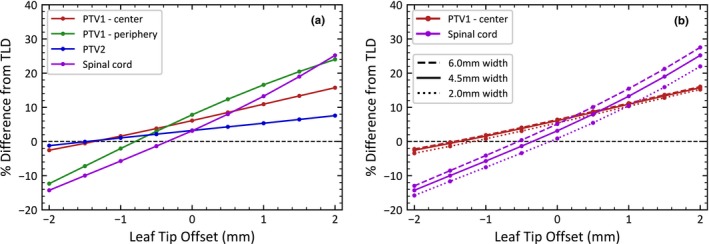
Percent difference in thermoluminesent dosimeter (TLD) dose reported by the treatment planning system (TPS) versus measured dose from the Imaging and Radiation Oncology Core Houston (IROC‐H) phantom as a function of leaf‐tip offset for (a) all TLDs with a 4.5 mm leaf‐tip width and (b) the PTV1 center and Spinal cord TLDs with various leaf‐tip widths.

Figure 3 shows the percent difference between the measured and calculated TLD doses as a function of leaf‐tip offset for (a) all TLDs, with a constant leaf‐tip width value of 4.5 mm, which matches the clinically commissioned model at our institution and (b) two TLDs with several leaf‐tip width values in order to investigate the effect of the leaf‐tip width. Note that these results represent one IMRT plan (IMRT0 in Table 1), which was the only plan with TLD measurements from the IROC‐H phantom. The dose agreement has a strong dependence on the leaf‐tip offset for all TLDs. Changing the leaf‐tip offset by only 1 mm can result in a dose difference of over 10% in some TLDs. The dependence on leaf‐tip offset is strongest in the Spinal cord and PTV1 periphery TLDs, both of which were in high dose gradient regions of the plan. The dependence on the leaf‐tip width was less pronounced, with a maximum variation of only 5.6% over the range of leaf‐tip widths for the Spinal cord TLD and less than 2% for all other TLDs. For this reason, the remainder of this study focuses only on the effects of the leaf‐tip offset, rather than the leaf‐tip width.

### 3.2 Effect of leaf‐tip offset on TLD agreement

**Figure 4:**
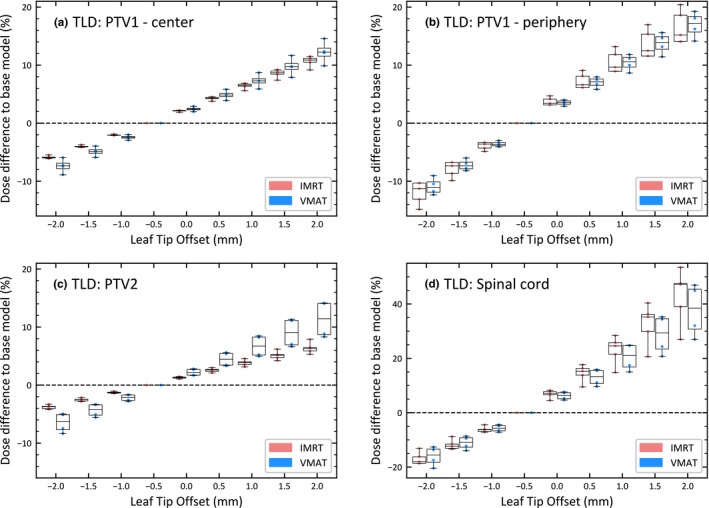
Percent difference in treatment planning system (TPS)‐calculated thermoluminesent dosimeter (TLD) dose between the adjusted model and the clinical model as a function of leaf‐tip offset for nine plans. TLDs at the following locations: (a) PTV1 center, (b) PTV1 periphery, (c) PTV2, and (d) Spinal cord. Note that the scale in (d) is different. The boxplots show the dose difference for five intensity‐modulated radiation therapy (IMRT) and four VMAT plans. Doses are compared to the clinical model (leaf‐tip offset at −0.5 mm). All leaf‐tip offsets correspond to those labeled on the x‐axis, though the data are staggered slightly for clarity.

Figure 4 shows the percent dose difference between the adjusted model and the clinical model as a function of leaf‐tip offset for all nine plans described in the methods. Note that the leaf‐tip width was held constant at 4.5 mm as described in the previous section. Each leaf‐tip offset shows two boxplots, one for IMRT (5 plans) and one for VMAT (4 plans). The clinical model, with a leaf‐tip offset of −0.5 mm, was used as a baseline. Thus, the dose difference at −0.5 mm leaf‐tip offset is equal to zero.

In general, a more negative leaf‐tip offset value, which corresponds to a smaller field size, underestimates the dose, while a more positive leaf‐tip offset, which corresponds to a larger field size, overestimates the dose. The results are approximately symmetric around the clinical model, though the exact relationship depends on the specific beam parameters of each plan. This can be seen in Figure 4 for all four TLDs, with models that have a leaf‐tip offset that is more negative than the clinical model underestimating the dose, and models with a more positive leaf‐tip offset overestimating the dose.

Similar to the results seen in Figure 3, the leaf‐tip offset has the largest effect on both the PTV1 periphery and Spinal cord TLDs, likely due to their location near high‐gradient regions of the plan. The responses between IMRT and VMAT follow the same trend, though the VMAT plans show greater variability between individual plans in the dose in low‐gradient regions, as seen in PTV1 — center and PTV2 TLD.

### 3.3 Detectability of model deficiencies by patient‐specific IMRT QA

**Figure 5:**
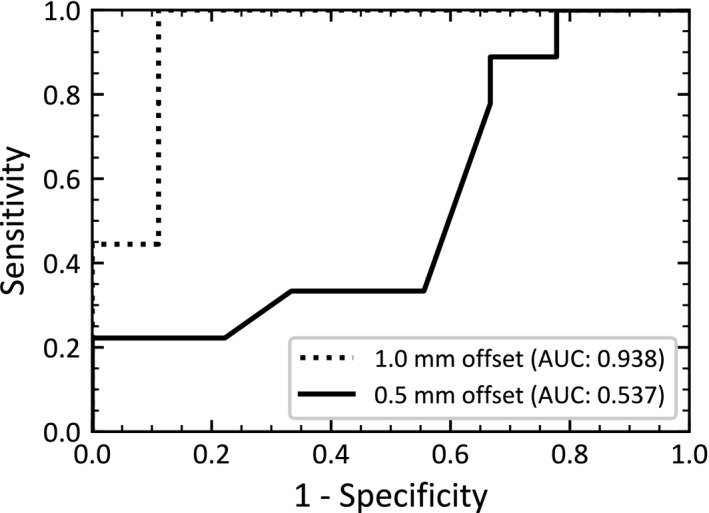
Receiver‐operating characteristic (ROC) curves showing the performance of patient‐specific intensity‐modulated radiation therapy (IMRT) quality assurance (QA) at detecting model failures (gamma criteria 3%/3 mm). The curve represents the ability of patient‐specific IMRT QA to distinguish between an acceptable model (i.e.: the clinical model) and an unacceptable model. Unacceptable model results are shown for 0.5 and 1.0 mm leaf‐tip offsets, which correspond to mean dose deviations of 7.2% and 11.1% across all plans and TLDs. See Figure 4 for further details on the dose deviation.

The detectability of the beam model variations was assessed by creating ROC curves varying the gamma pass rate (Figure 5). The curve represents the ability of patient‐specific IMRT QA to distinguish between an acceptable model (i.e.: the clinical model) and an unacceptable model. AUCs of 0.537 and 0.938 were found for 0.5 and 1.0 mm leaf‐tip offsets, which correspond to mean dose deviations of 7.2% and 11.1% across all plans and TLDs. While patient‐specific IMRT QA performs well for large dose deviations (AUC 0.938 for 1 mm offset), it is inadequate at detecting models with small to moderate dose deviations. These results were nearly identical when comparing IMRT and VMAT, as well as when comparing 2%/2 mm and 3%/3 mm dose criteria.

## 4 DISCUSSION

This study investigated the dosimetric effects of MLC beam model parameters in the context of IMRT and VMAT planning in a commercial TPS. Small changes in the MLC leaf‐tip offset had large effects on the dose. A 1 mm change in the leaf‐tip offset led to dose deviations of up to 10% in the PTV and 15% outside the PTV. These differences were detected neither through the commissioning process nor through standard TPS validation tests advocated by professional societies. Patient‐specific IMRT QA was also unable to reliably detect model changes except in situations of very large dose differences. Using restricted criteria (e.g. 2%/2 mm) also did not improve the detectability. Only external validation through the IROC‐H phantom was able to detect the modeling errors in this case.

A number of previous studies have also noted a strong dependence of the IMRT dose calculation with the position of the MLC leaves.^10^, ^13^, ^14^, ^15^, ^16^, ^17^, ^18^, ^19^, ^20^, ^21^ This study, however, uses a different MLC model and TPS, as well as step‐and‐shoot IMRT and VMAT delivery. It also considers the detectability of deviations in these parameters through patient‐specific QA and anthropomorphic phantoms. The TPS considered here does not use full ray‐tracing. Instead, the MLC leaf‐tip width and offset are used to model the rounded ends of the MLC leaves. While variations in these parameters have minimal effect on the open field profiles and depth dose curves, they have large effects on the calculated dose in IMRT and VMAT plans, leading to potential for errors.

Some previous studies have focused on miscalibration of the MLC and the potential dosimetric impacts.^17^, ^18^ Cadman et al.^15^, ^16^ found large dose dependences with the MLC leaf position in early versions of the Pinnacle planning system. However, the bulk of the literature has examined the impact of the leaf model in the Eclipse TPS. The Eclipse system uses a dosimetric leaf gap (DLG) parameter to describe the offset of the MLC leaves, defined as the difference between the 50% radiological field width and the nominal radiological field size.^20^ While the parameters are not identical, Eclipse's DLG and RayStation's leaf‐tip offset both represent shifts of the MLC from its nominal position,^20^ allowing comparisons to be drawn between this study and DLG‐based studies. The studies of the Eclipse model suggest that small changes in the MLC offset can have large effects on the dose calculation for IMRT and VMAT plans. Lee et al.^19^ found that a change in the DLG of 1 mm caused large (>10%) changes in the calculated dose. Kielar et al.^20^ also found that varying the DLG by 1 mm from the optimal value determined during commissioning led to a 5% difference between measured and calculated doses in clinical treatment plans. The results here are consistent with these findings and extend the results to address the question of whether such deviations are detectable with patient‐specific IMRT QA and external audits.

While a few publications have also investigated the RayStation beam model, to our knowledge no study to date has systematically investigated the impact of the leaf‐tip offset. Chen et al.^26^ optimized the MLC model, varying the MLC leaf‐tip width, MLC transmission factor, and tongue‐and‐groove width for Varian linacs. In their study, the leaf‐tip offset was set per vendor recommendations, and its impact was not investigated. Mzenda et al.^27^ modeled both the Elekta MLCi and Agility collimators. Though they did not explore model dependencies, they found excellent model agreement with IMRT QA and dose curve comparison. Dose measured inside an anthropomorphic thorax phantom with a 1 cm spherical target was within 1% of the TPS. However, no measurements were taken outside of the target, a region with the greatest variability in the current study.

Despite significant evidence in the literature that patient‐specific IMRT QA fails to detect TPS errors,^9^, ^10^, ^11^, ^12^ many institutions rely on it for TPS commissioning. It is advocated by AAPM MPPG 5.a^1^ and TG 119^22^ as one of the key methods to validate commissioning of TPS, though with caveats on detector size and spacing that are often overlooked. The results here support and supplement the body of evidence in the literature, showing that patient‐specific IMRT QA cannot reliably identify inferior MLC beam models unless the dose deviation is very large (>10%). Carlone et al.^9^ introduced random shifts to the MLC leaves and found that IMRT QA was only sensitive to very large errors (>3 mm shifts). Kruse^11^ showed that gamma analysis was insensitive to inaccurate dose calculation by comparing ion chamber measurements to IMRT QA using gamma analysis. Nelms et al.^12^ found no correlation between gamma pass rates and errors introduced to the penumbra and MLC transmission parameters in the beam model. Interestingly, the gamma criteria chosen (2%/2 mm or 3%/3 mm) had little effect on the quality of the test, a result that also agrees with recent findings in the literature.^8^, ^12^ The results presented here extend these findings of limitations in QA with the explicit goal of tying them to dose deviations in the treatment plan caused by variations in beam model parameters. The results presented here are also consistent with Kry et al.,^8^ who showed that IMRT QA provides no predictive power for failures of the IROC‐H phantom‐based tests.

Some parameters within the TPS were not studied here, such as the MLC transmission, tongue and groove effect, and the gain and curvature of MLC leaf‐tip position. These are reported elsewhere^26^, ^27^ and were not found to have a large impact on IMRT dose calculation at our institution. Only Elekta linacs with the Agility collimator and a 6 MV photon beam were studied. It is possible that these results may be different for MLC systems with different geometric parameters — such as radius of curvature of the rounded leaf ends, which are not modeled with the simple step functions — or through a more sophisticated ray‐tracing treatment of the MLC. Finally, all results presented here are based on the IROC‐H head‐and‐neck phantom with 6 MV photons. Though this choice may be justified by the fact that MLC leaf‐tip offsets are thought to have a stronger effect in plans with larger dose gradients,^14^ it may also be valuable to investigate other anthropomorphic phantoms, such as prostate, lung, liver, or SRS phantoms. The collection of multi‐institutional data through increased use of the IROC‐H program may provide additional insight; according to Glenn et al.,^28^ as of December 2016, only nine institutions using RayStation had participated in the IROC‐H head‐and‐neck phantom irradiation program, and only four of these used Elekta linacs.

## 5 CONCLUSIONS

The MLC modeling parameters in a commercial TPS were investigated in the context of IMRT treatment planning. Changes in these parameters had large effects on IMRT dose, but these effects were not readily apparent during the standard modeling, commissioning, and validation processes. This effect was much more pronounced for the leaf‐tip offset than the leaf‐tip width, with dose differences up to 20% for a 1 mm shift in the MLCs. IMRT QA was unable to detect failing models unless the dose deviation was very large (>20%). Only external validation with an anthropomorphic phantom was able to reliably detect failing models. Care should be taken during the modeling process of the MLCs in a new planning system, and external audits recommended by national and international societies are an essential component of safe TPS commissioning.

## CONFLICT OF INTEREST

No conflicts of interest.

## References

[1] Smilowitz JB , Das IJ , Feygelman V , et al. Medical Physics Practice Guideline 5.a.: commissioning and QA of treatment planning dose calculations — Megavoltage photon and electron beams. J Appl Clin Med Phys. 2015;16:14–34. 26699330 10.1120/jacmp.v16i5.5768 PMC5690154

[2] Donaldson SL , The Royal College of Radiologists , Society and College of Radiographers , Institute of Physics and Engineering in Medicine , National Patient Safety Agency , British Institute of Radiology . Towards Safer Radiotherapy. London: The Royal College of Radiologists; 2008.

[3] Zietman AL , Palta JR , Steinberg ML . Safety is no accident: a framework for quality radiation oncology and care. Am Soc Radiat Oncol. 2012.

[4] Moran JM , Dempsey M , Eisbruch A , et al. Safety considerations for IMRT: executive summary. Pract Radiat Oncol. 2011;1:190–195. 25740119 10.1016/j.prro.2011.04.008 PMC3808751

[5] Palta JR , Deye JA , Ibbott GS , et al. Credentialing of institutions for IMRT in clinical trials. Int J Radiat Oncol Biol Phys. 2004;59:1257–1261. 15234063 10.1016/j.ijrobp.2004.03.007

[6] Kerns JR , Followill DS , Lowenstein J , et al. Agreement between institutional measurements and treatment planning system calculations for basic dosimetric parameters as measured by the imaging and radiation oncology core‐houston. Int J Radiat Oncol Biol Phys. 2016;95:1527–1534. 27315667 10.1016/j.ijrobp.2016.03.035 PMC5113287

[7] Kerns JR , Stingo F , Followill DS , et al. Treatment planning system calculation errors are present in most imaging and radiation oncology core‐houston phantom failures. Int J Radiat Oncol Biol Phys. 2017;98:1197–1203. 28721904 10.1016/j.ijrobp.2017.03.049 PMC5567850

[8] Kry SF , Molineu A , Kerns JR , et al. Institutional patient‐specific IMRT QA does not predict unacceptable plan delivery. Int J Radiat Oncol Biol Phys. 2014;90:1195–1201. 25442044 10.1016/j.ijrobp.2014.08.334 PMC4276500

[9] Carlone M , Cruje C , Rangel A , et al. ROC analysis in patient specific quality assurance. Med Phys Med Phys. 2013;40:42103–4530. 10.1118/1.4795757 23556913

[10] Nelms BE , Chan MF , Jarry G , et al. Evaluating IMRT and VMAT dose accuracy: practical examples of failure to detect systematic errors when applying a commonly used metric and action levels. Med Phys. 2013;40:111722. 24320430 10.1118/1.4826166 PMC8353583

[11] Kruse JJ . On the insensitivity of single field planar dosimetry to IMRT inaccuracies. Med Phys. 2010;37:2516–2524. 20632563 10.1118/1.3425781

[12] Nelms BE , Zhen H , Toḿ WA . Per‐beam, planar IMRT QA passing rates do not predict clinically relevant patient dose errors. Med Phys. 2011;38:1037–1044. 21452741 10.1118/1.3544657 PMC3188652

[13] LoSasso T , Chui CS , Ling CC . Physical and dosimetric aspects of a multileaf collimation system used in the dynamic mode for implementing intensity modulated radiotherapy. Med Phys. 1998;25:1919–1927. 9800699 10.1118/1.598381

[14] Budgell GJ , Mott JHL , Williams PC , et al. Requirements for leaf position accuracy for dynamic multileaf collimation. Phys Med Biol. 2000;45:1211–1227. 10843101 10.1088/0031-9155/45/5/310

[15] Cadman P , Bassalow R , Sidhu NPS , et al. Dosimetric considerations for validation of a sequential IMRT process with a commercial treatment planning system. Med Biol Phys Med Biol. 2002;47:3001–3010. 12222862 10.1088/0031-9155/47/16/314

[16] Cadman P , McNutt T , Bzdusek K . Validation of physics improvements for IMRT with a commercial treatment‐planning system. J Appl Clin Med Phys. 2005;6:74–86. 15940214 10.1120/jacmp.v6i2.2083 PMC5723472

[17] Rangel A , Dunscombe P . Tolerances on MLC leaf position accuracy for IMRT delivery with a dynamic MLC. Med Phys. 2009;36:3304–3309. 19673226 10.1118/1.3134244

[18] Luo W , Li J , Price RA , et al. Monte Carlo based IMRT dose verification using MLC log files and R/V outputs. Med Phys. 2006;33:2557–2564. 16898460 10.1118/1.2208916

[19] Lee J‐W , Choi K‐S , Hong S , et al. Effects of static dosimetric leaf gap on MLC‐based small beam dose distribution for intensity modulated radiosurgery. J Appl Clin Med Phys. 2007;8:54–64. 10.1120/jacmp.v8i4.2397 PMC5722628 18449146

[20] Kielar KN , Mok E , Hsu A , et al. Verification of dosimetric accuracy on the TrueBeam STx: rounded leaf effect of the high definition MLC. Med Phys. 2012;39:6360–6371. 23039672 10.1118/1.4752444

[21] Yao W , Farr JB . Determining the optimal dosimetric leaf gap setting for rounded leaf‐end multileaf collimator systems by simple test fields. J Appl Clin Med Phys. 2015;16:65–77. 10.1120/jacmp.v16i4.5321 PMC5690020 26218999

[22] Ezzell GA , Burmeister JW , Dogan N , et al. TG 119: IMRT commissioning: Multiple institution planning and dosimetry comparisons, a report from AAPM Task Group 119. Med Phys. 2009;36:5359–5373. 19994544 10.1118/1.3238104

[23] Molineu A , Followill DS , Balter PA , et al. Design and implementation of an anthropomorphic quality assurance phantom for intensity‐modulated radiation therapy for the Radiation Therapy Oncology Group. Int J Radiat Oncol Biol Phys. 2005;63:577–583. 16168849 10.1016/j.ijrobp.2005.05.021

[24] Miften M , Olch A , Mihailidis D , et al. TG 218: Tolerance limits and methodologies for IMRT measurement‐based verification QA: recommendations of AAPM Task Group No. 218. 2018. 10.1002/mp.12810 29443390

[25] Bojechko C , Ford EC . Quantifying the performance of in vivo portal dosimetry in detecting four types of treatment parameter variations. Med Phys. 2015;42:6912–6918. 26632047 10.1118/1.4935093

[26] Chen S , Yi BY , Yang X , et al. Optimizing the MLC model parameters for IMRT in the RayStation treatment planning system. J Appl Clin Med Phys. 2015;16:322–332. 10.1120/jacmp.v16i5.5548 PMC5690186 26699315

[27] Mzenda B , Mugabe KV , Sims R , et al. Modeling and dosimetric performance evaluation of the RayStation treatment planning system. J Appl Clin Med Phys. 2014;15:29–46. 10.1120/jacmp.v15i5.4787 PMC5711080 25207563

[28] Glenn MC , Hernandez V , Saez J , et al. Treatment plan complexity does not predict IROC Houston anthropomorphic head and neck phantom performance. Phys Med Biol. 2018;63:205015. 30230475 10.1088/1361-6560/aae29e PMC6287268

